# AAV-RPGR Gene Therapy Rescues Opsin Mislocalisation in a Human Retinal Organoid Model of *RPGR*-Associated X-Linked Retinitis Pigmentosa

**DOI:** 10.3390/ijms25031839

**Published:** 2024-02-02

**Authors:** Paul E. Sladen, Arifa Naeem, Toyin Adefila-Ideozu, Tijmen Vermeule, Sophie L. Busson, Michel Michaelides, Stuart Naylor, Alexandria Forbes, Amelia Lane, Anastasios Georgiadis

**Affiliations:** 1 MeiraGTx UK II, 34-38 Provost Street, London N1 7NG, UKamelia.lane@meiragtx.com (A.L.); 2 Moorfields Eye Hospital, 162 City Road, London EC1V 2PD, UK; 3 University College London Institute of Ophthalmology, London EC1V 9LF, UK

**Keywords:** RPGR, retinitis pigmentosa, X-linked, gene therapy, adeno associated virus, IPSC, retinal organoids, CRISPR/Cas9

## Abstract

Variants within the Retinitis Pigmentosa GTPase regulator (*RPGR*) gene are the predominant cause of X-Linked Retinitis Pigmentosa (XLRP), a common and severe form of inherited retinal disease. XLRP is characterised by the progressive degeneration and loss of photoreceptors, leading to visual loss and, ultimately, bilateral blindness. Unfortunately, there are no effective approved treatments for RPGR-associated XLRP. We sought to investigate the efficacy of RPGR^ORF15^ gene supplementation using a clinically relevant construct in human RPGR-deficient retinal organoids (ROs). Isogenic RPGR knockout (KO)-induced pluripotent stem cells (IPSCs) were generated using established CRISPR/Cas9 gene editing methods targeting *RPGR*. RPGR-KO and isogenic wild-type IPSCs were differentiated into ROs and utilised to test the adeno associated virus (AAV) RPGR (AAV-RPGR) clinical vector construct. The transduction of RPGR-KO ROs using AAV-RPGR successfully restored RPGR mRNA and protein expression and localisation to the photoreceptor connecting cilium in rod and cone photoreceptors. Vector-derived RPGR demonstrated equivalent levels of glutamylation to WT ROs. In addition, treatment with AAV-RPGR restored rhodopsin localisation within RPGR-KO ROs, reducing mislocalisation to the photoreceptor outer nuclear layer. These data provide mechanistic insights into RPGR^ORF15^ gene supplementation functional potency in human photoreceptor cells and support the previously reported Phase I/II trial positive results using this vector construct in patients with RPGR-associated XLRP, which is currently being tested in a Phase III clinical trial.

## 1. Introduction

Variants in the Retinitis Pigmentosa GTPase regulator (*RPGR*) gene are the predominant cause of X-Linked Retinitis Pigmentosa (XLRP), a severe form of inherited retinal disease (IRD) [1]. XLRP is characterised by the progressive degeneration and loss of photoreceptors, leading to visual loss and, ultimately, bilateral blindness [1,2]. The *RPGR* gene has two primary isoforms: constitutive *RPGR* encoded by exons 1–19 (*RPGR^1−19^*) that is expressed at the transition zone in the majority of ciliated tissues, and *RPGR^ORF15^*, the major isoform expressed in photoreceptor cells. *RPGR^1−19^* and *RPGR^ORF15^* share the first 14 exons [3,4], with *ORF15* extending into *RPGR* intron 15 and encoding a glutamic-acid- and glycine-rich domain. *RPGR^ORF15^* is a variant hotspot, accounting for approximately 60% of XLRP cases [5], with the majority of variants causing frameshift or a premature termination codon (PTC) [6,7,8]. Treatments for *RPGR*-associated XLRP are currently non-existent.

RPGR^ORF15^ locates to the photoreceptor connecting cilium (CC), a structure that links and acts as a gateway for protein trafficking between the photoreceptor biosynthetic inner segment (IS) and the light-sensing outer segment (OS). The transport of proteins, including the opsins, through the CC is tightly regulated and its disturbance is implicated in many IRDs, including *RPGR*-linked XLRP [9].

The glutamylation of RPGR^ORF15^, within the glutamic-acid- and glycine-rich domain, is a post-translational modification that has been demonstrated to be directly linked to RPGR function [10,11]. Previously, it has been shown that RPGR-deficient mouse models have a reduction in polyglutamylation at the photoreceptor CC, resulting in opsin mislocalisation within the IS and outer plexiform layer [10,12]. Three dimensional (3D) retinal organoid (RO) models derived from patients or with engineered gene variants have proven valuable in both disease modelling and preclinical drug development [13,14,15,16,17,18]. Previously, ROs derived from patients with *RPGR* XLRP have been shown to have reduced glutamylation at the CC and mislocalisation of opsin proteins [19,20,21].

Here, we sought to test the potency and function of an adeno associated virus (AAV) RPGR^OF15^ vector, AAV7m8-hRKp.RPGR (AAV-RPGR), using an RO model of *RPGR-*associated XLRP. This vector comprises the human rhodopsin kinase promoter (hRKp) *RPGR* construct (utilised in Botaretigene Sparoparvovec in the phase III Lumeos XLRP clinical trial [22]), packaged into a AAV7m8 capsid, which is a more efficient transducer of human ROs during the early photoreceptor developmental stages [23,24]. Following the transduction of CRISPR/Cas9 engineered RPGR-KO ROs, vector potency was assessed by comparing RPGR protein, mRNA, glutamylation and the localisation of opsin relative to isogenic wild-type (WT) ROs derived from the same genetic background. These data corroborate previously reported Phase I/II trial positive results in patients with *RPGR*-associated XLRP [22] and provide detailed mechanistic insights into vector potency in human photoreceptor cells.

## 2. Results

### 2.1. Generation of RPGR-KO IPSCs

Simultaneous cell reprogramming and CRISPR/Cas9 gene editing were utilised to generate an isogenic RPGR-KO IPSC line by inducing insertion and deletion variants in *RPGR* into an otherwise healthy human fibroblast cell line. *RPGR* exon 10, a clinically relevant *RPGR* exon [25,26,27,28], was targeted with two CRISPR/Cas9 gRNAs, with the induction of a 131 base pair (bp) deletion confirmed via Sanger sequencing (Figure S1A). In silico analysis of *RPGR* mRNA transcripts harbouring the 131 bp deletion predicted the induction of downstream PTCs within *RPGR* exon 11 (Figure S1B). An analysis of the expression of stem cell pluripotency markers confirmed the expression of LIN28, SOX2 and OCT4 within RPGR-KO IPSCs (Figure S1C), suggesting the induction of endogenous self-renewal programs and the generation of bona fide IPSCs.

### 2.2. Characterisation of RPGR-KO ROs

**Figure 1:**
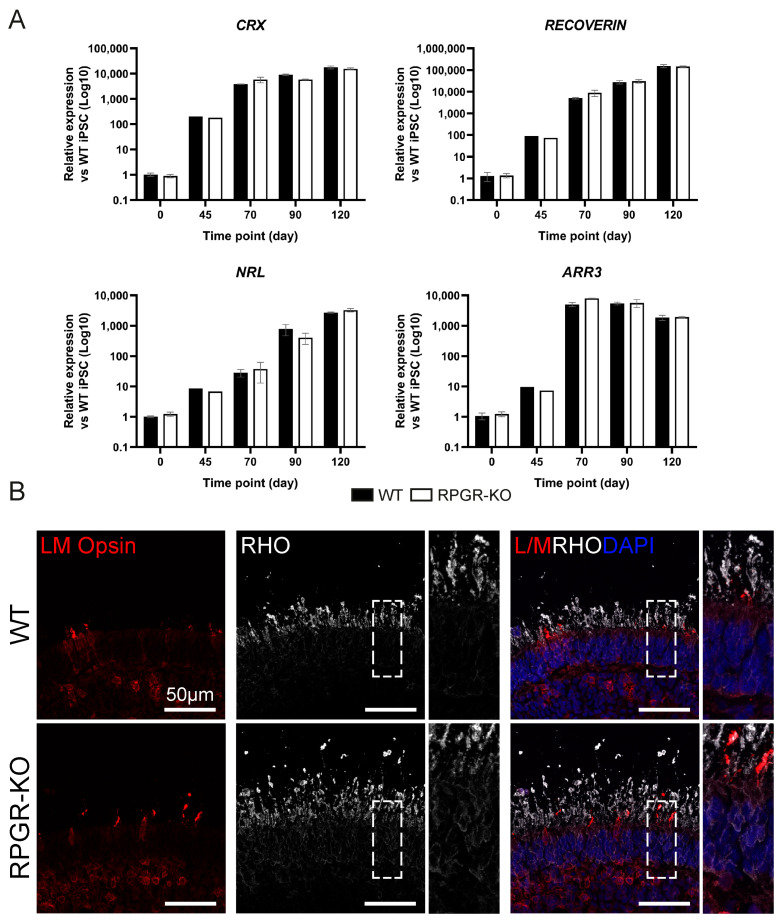
Assessment of RPGR-KO RO differentiation capacity. (**A**) WT and RPGR-KO d120 ROs express key retinal genes CRX, RECOVERIN, NRL and ARR3. Gene expression was first normalised to the geometric mean of GAPDH and ACTIN, then to d0 naive IPSCs. *n* = 1–4 ROs. (**B**) L/M Opsin (red) and Rhodopsin (RHO; white) expression in d160 WT and RPGR-KO ROs. Nuclei are identified with DAPI (blue). Zoomed panel denoted by dashed box. Scale bar = 50 μm.

RPGR-KO IPSCs were differentiated towards a 3D RO fate following established methods [20]. A quantitative PCR (QPCR) analysis of WT and RPGR-KO ROs at d120 demonstrated the expression of key retinal markers *CRX*, *RECOVERIN*, *NRL* and *ARR3*, with no significant differences detected between either cell line (Figure 1A). An immunocytochemistry (ICC) analysis confirmed the expression of L/M Opsin and Rhodopsin at d160 of differentiation (Figure 1B), confirming the successful differentiation of ROs from RPGR-KO IPSCs. During this initial characterisation of RPGR-KO ROs, we detected signs of Rhodopsin mislocalisation within the RPGR-KO ONL (Figure 1B), highlighting this as a potential disease phenotype within RPGR-KO ROs. Previous studies of mice retina and human ROs have demonstrated opsin mislocalisation [12,20,21].

**Figure 2:**
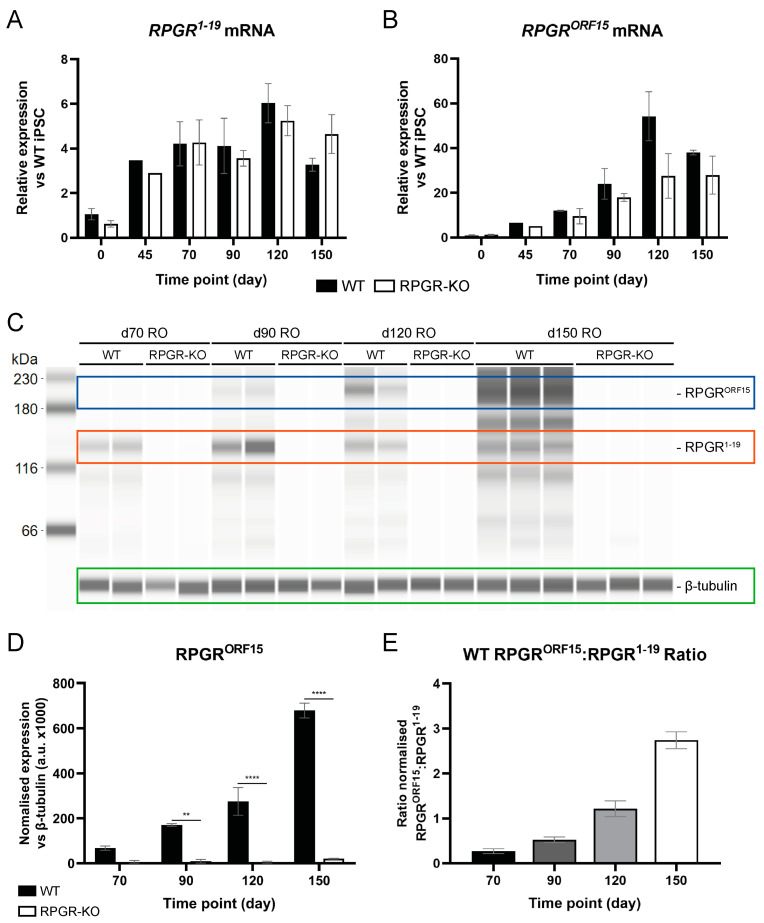
Characterisation of RPGR expression during retinal organoid development. (**A**,**B**) Analysis of RGPR isoform expression during RO development. *RPGR* isoform expression was first normalised to the geometric mean of *GAPDH* and *ACTIN*, then to d0 naive IPSCs. *n* = 1–4 ROs. (**C**) Immunoblotting of RPGR expression during RO development. RPGR^1−19^ (red box) is expressed at 140 kDa; RPGR^ORF15^ (blue box) is detected at 200 kDa. Loading control β-tubulin (green box) is detected at 55 kDa. One organoid per lane. (**D**) Immunoblotting quantification demonstrates a significant reduction in RPGRORF15 protein in RPGR-KO ROs when compared to WT ROs. *n* = 2–4 ROs, ** *p* < 0.01, **** *p* < 0.0001. (**E**) RPGR^119^:RPGR^ORF15^ ratio during WT RO differentiation.

Subsequently, we sought to establish the expression of RPGR isoforms throughout RO differentiation. QPCR primers were designed to target two distinct regions of *RPGR* to enable the quantification of expression of mRNA from *RPGR^1−19^* and *RPGR^ORF15^* isoforms. Following induction, relative *RPGR^1−19^* mRNA expression levels remained stable from d45–d150, at a four-to-six-fold change when compared to naïve IPSCs (Figure 2A). In contrast, *RPGR^ORF15^* increased significantly over the time-course of retinogenesis (Figure 2B), with a 40–60-fold change compared to d0 IPSCs, thus demonstrating the recapitulation of retina-specific alternative splicing of *RPGR* in the RO model. RPGR-KO ROs demonstrated a reduction in *RPGR* mRNA for the distinct RPGR isoforms, but no statistical differences between WT and RPGR-KO ROs were detected (Figure 2A,B). To establish why RPGR-KO ROs retained *RPGR* mRNA, reverse transcriptase-PCR (RT-PCR) was completed, analysing the alternative splicing of exons surrounding *RPGR* exon 10, given the fact that the 131 bp deletion induces downstream premature termination codons in exon 11. RT-PCR analysis confirmed the presence of *RPGR* mRNA in both WT and RPGR-KO ROs at all time-points analysed (Figure S2A). However, RPGR-KO ROs demonstrated a notably smaller RT-PCR product size when compared to WT ROs (Figure S2A), indicative of *RPGR* exon 10 skipping within RPGR-KO ROs.

Following the analysis of *RPGR* mRNA, RPGR protein levels were quantified by immunoblotting. Within WT ROs, immunoblotting analysis demonstrated a temporal induction of the retinal-specific RPGR^ORF15^ isoform (MW 210 kDa), with maximal expression identified at d150 (Figure 2C,D). RPGR^1−19^ (MW 140 kDa) demonstrated consistent expression throughout retinogenesis (Figure S2B). Immunoblotting confirmed the absence of RPGR protein in RPGR-KO ROs when compared to WT ROs (Figure 2C,D), confirming the generation of RPGR-deficient ROs. The ratio of RPGR^ORF15^ to RPGR^1−19^ for WT organoids showed a significant shift in RPGR expression toward the RPGR^ORF15^ isoform throughout the RO differentiation time course (Figure 2E). Interestingly, the RPGR antibody, which targets RPGR amino acids 379 to 509, detects additional bands ranging in size from 110 to 210 kDa, all of which were absent in RPGR-KO ROs. This is in keeping with the presence of alternately sized RPGR isoforms in the WT, as reported previously [10,12].

### 2.3. Characterisation of RPGR-KO ROs Treated with AAV-RPGR

**Figure 3:**
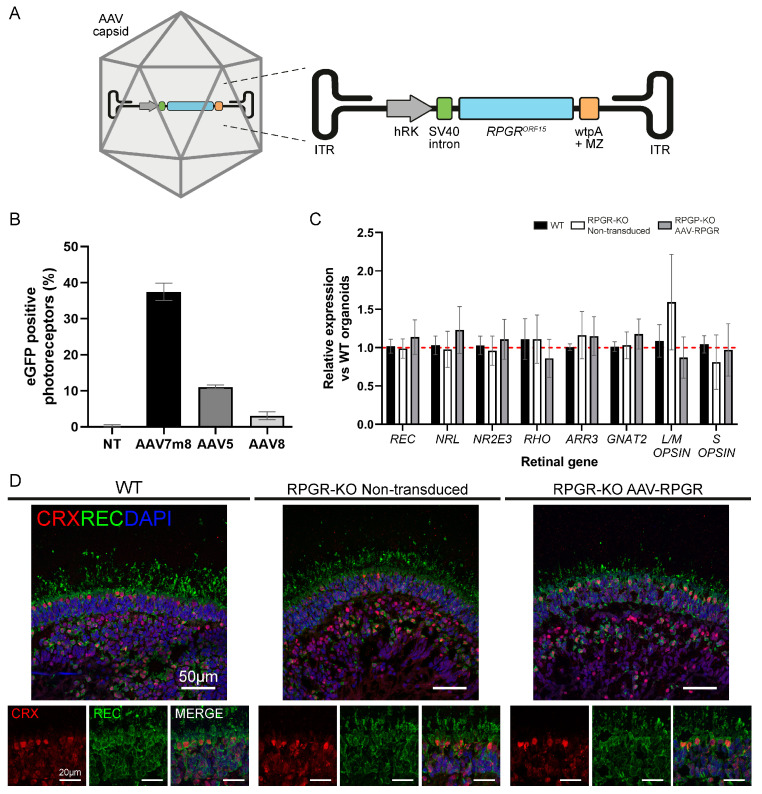
Characterisation of AAV-RPGR treated ROs. (**A**) Schematic representation of AAV-RPGR. (**B**) Flow cytometry analysis of AAV capsid RO transduction efficiency. ROs were transduced with an eGFP transgene vector packaged in AAV7m8, AAV5 or AAV8. *n* = 4 ROs. (**C**) RPGR deficiency and AAV-RPGR treatment have no effect on retina-associated gene expression in d160 ROs. Gene expression was normalised to the geometric mean of *GAPDH* and *ACTIN*, and then to WT ROs. Red dashed line demarcates normalised WT expression levels. *n* = 3–6 ROs. (**D**) WT, non-transduced and AAV-RPGR transduced RPGR-KO d160 ROs express CRX (red) and RECOVERIN (REC; green). Nuclei are identified with DAPI (blue). Main image scale bar = 50 μm. Panel image scale bar = 20 μm.

Following RPGR-KO RO characterisation, RPGR-KO ROs were transduced with AAV-RPGR, carrying the RPGR^ORF15^ clinical construct (Figure 3A), at d135. Of note, ROs require high doses for efficient transduction [15,19], and consequently we packaged the RPGR^ORF15^ clinical transgene into AAV7m8, which has enhanced transduction efficiency in developmental RO photoreceptors relative to the AAV5 and AAV8 capsids (Figure 3B). Following transduction, ROs were incubated for a further 25 days of culture and isolated at d160 for the analysis of retinal morphology, RPGR expression and rescue of RPGR deficiency phenotypes.

The initial analysis of RO retinal gene expression, including *NRL*, *ARR3* and *L/M-OPSIN* (*OPN1LW*/*OPN1MW*), confirmed no significant detrimental effects of AAV-RPGR transduction on RPGR-KO ROs when compared to untreated RPGR-KO ROs or WT ROs (Figure 3C). ICC staining confirmed the presence of CRX-positive photoreceptor cells within the RO outer nuclear layer (ONL), with the extension of RECOVERIN positive OS into the peripheral space (Figure 3D). No morphological differences were detected between WT, non-transduced and AAV-RPGR-treated ROs (Figure 3D).

**Figure 4:**
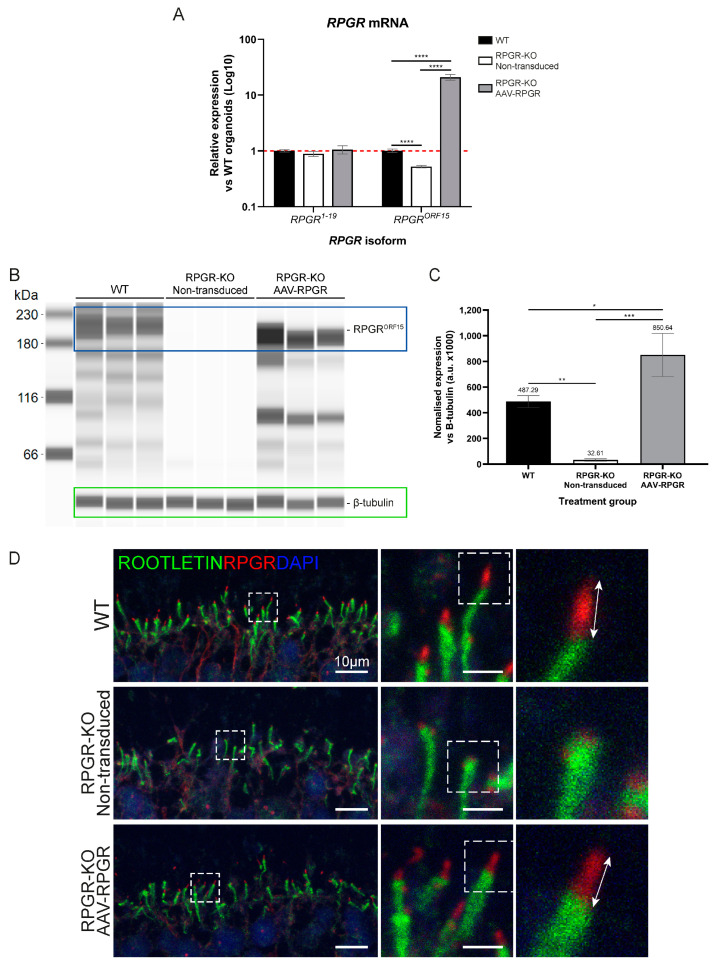
AAV-RPGR restores RPGR expression and localisation in RPGR-KO ROs. (**A**) AAV-RPGR treatment significantly increases *RPGR^ORF15^* expression in RGPR-KO ROs. Gene expression was normalised to the geometric mean of *GAPDH* and *ACTIN*, and then to WT ROs. Red dashed line demarcates normalised WT expression levels. *n* = 3–6 ROs. **** *p* < 0.0001. (**B**) AAV-RPGR treatment restores RPGR^ORF15^ (185 kDa, blue box) expression in d160 RPGR-KO ROs. Loading control β-tubulin (green box) is detected at 55 kDa. One organoid per lane. (**C**) Immunoblotting quantification demonstrates significantly increased RPGR^ORF15^ protein levels in RPGR-KO ROs following AAV-RPGR transduction. *n* = 3–4 ROs. * *p* < 0.05, ** *p* < 0.01, *** *p* < 0.001. (**D**) RPGR localisation in the RO connecting cilium. WT ROs express RPGR protein (red) at the apical tip of the ciliary rootlet (ROOTLETIN; green). RPGR-KO ROs demonstrate mislocalised RPGR within the ciliary rootlet. AAV-RPGR transduction restores RPGR localisation in RPGR-KO ROs. Zoomed panels denoted by dashed boxes. Cell nuclei are identified with DAPI (blue). Arrows highlight the region of correct RPGR localisation. Main image scale bar = 10 μm, zoom image scale bar = 2 μm.

Subsequently, we sought to confirm the effect of AAV-RPGR transduction on the expression of RPGR. RPGR-KO ROs demonstrated significantly reduced levels of *RPGR^ORF15^* when compared to isogenic WT ROs (Figure 4A). AAV-RPGR transduced RPGR-KO ROs demonstrated significantly upregulated expression of the *RPGR^ORF15^* transcript when compared to WT and untreated RPGR-KO ROs (Figure 4A). *RPGR^1−19^* showed no changes in expression, an expected result given that AAV-RPGR does not encode the *RPGR^1−19^* isoform (Figure 4A). Immunoblotting of d160 ROs demonstrated the absence of RPGR^ORF15^ protein in RPGR-KO samples (Figure 4B,C). Three weeks post-transduction, RPGR-KO ROs demonstrated a restoration of RPGR^ORF15^ (MW 185 kDa), the smaller size reflecting the shortened ORF15 domain in the AAV-RPGR transgene (Figure 4B,C). In addition, bands at lower molecular weights were visible in the transduced ROs, suggesting the presence of alternately processed vector-derived RPGR. Importantly, these bands were also detected in the WT organoids with this antibody (targeting RPGR amino acids 379–509).

Following the confirmation of RPGR transgene expression, we used ICC analysis to confirm the correct localisation of RPGR^ORF15^ to the photoreceptor CC. WT ROs demonstrated expression of RPGR at the apical tip of the ciliary rootlet (Figure 4D), in keeping with previous studies [10,12,21,29]. Some residual signal was detected in the RPGR-KO RO periphery, but this appeared fainter and mislocalised relative to WT ROs, overlapping with ROOTLETIN expression (Figure 4D). Transduction with AAV-RPGR rescued RPGR expression in RPGR-KO ROs, restoring localisation to the tip of the ciliary rootlet (Figure 4D). Collectively, these data confirm the successful transduction of RPGR-KO ROs and the robust expression of vector-derived RPGR.

### 2.4. Restoration of Glutamylated RPGR and Rhodopsin Localisation in RPGR-KO Retinal Organoids following AAV-RPGR Transduction

**Figure 5:**
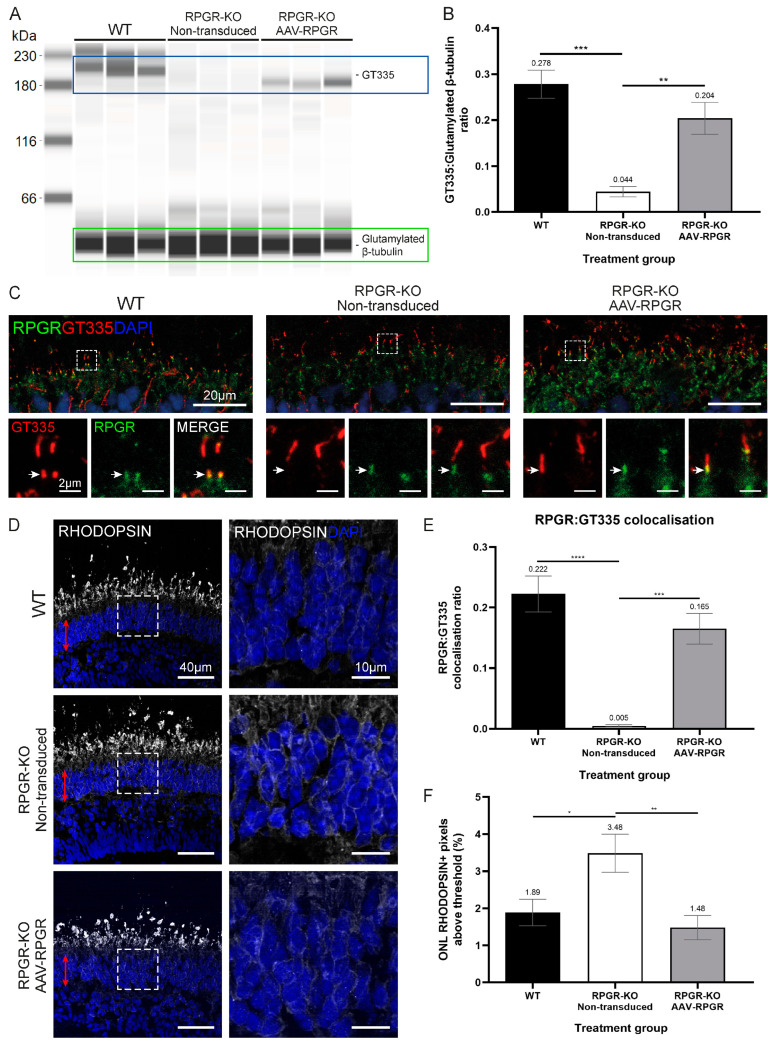
Restoration of RPGR glutamylation and rhodopsin localisation in RPGR-KO ROs by AAV-RPGR. (**A**) Immunoblotting analysis of RPGR glutamylation (GT335, blue box) in WT, non-transduced and AAV-RPGR-treated RPGR-KO organoids. Glutamylated β-tubulin (green box) is detected at 55 kDa. One organoid per lane. (**B**) Quantification of immunoblotting RPGR glutamylation levels, calculated as RPGR/β-tubulin ratio, demonstrates increased RPGR glutamylation in AAV-RPGR-treated RPGR-KO ROs compared to non-transduced ROs. *n* = 3–6 ROs, ** *p* < 0.01, *** *p* < 0.001. (**C**) ICC analysis of RGPR:GT335 co-localisation in d160 WT, non-transduced RPGR-KO and AAV-RPGR-treated RPGR-KO ROs. Arrows highlight areas of co-localisation between GT335 and RPGR in WT and AAV-RPGR treated ROs. Zoomed panel denoted by dashed box. Main image scale bar = 20 μm, zoom image scale bar = 2 μm. (**D**) ICC analysis of rhodopsin (white) mislocalisation in d160 WT, non-transduced RPGR-KO and AAV-RPGR-treated RPGR-KO ROs. Nuclei identified using DAPI (blue). Retina outer nuclear layer (ONL) denoted by red arrows. Zoomed panel denoted by dashed box. Scale bar = 40 μm, zoom scale bar = 10 μm. (**E**) Quantification of RPGR:GT335 co-localisation. *n* = 2–3 ROs. *** *p* < 0.001, **** *p* < 0.0001. (**F**) Quantification of rhodopsin mislocalisation, calculated as percentage of pixels above threshold in the ONL compared to total rhodopsin pixels. *n* = 2–3 ROs. * *p* < 0.05, ** *p* < 0.01.

Post-translational glutamylation of RPGR^ORF15^ has been shown to critically affect its function in photoreceptors [10,11]. To determine if the transduction of RPGR-KO ROs with AAV-RPGR caused the rescue of RPGR-variant-associated characteristics, we quantified RPGR glutamylation levels using GT335, an antibody reactive to (poly)-glutamylation post-translational modifications on both RPGR and tubulin proteins [10,11]. Immunoblotting demonstrated a loss of glutamylated protein at 185 kDa within RPGR-KO ROs when compared to WT ROs (Figure 5A,B). The transduction of RPGR-KO ROs with AAV-RPGR demonstrated a significant restoration of glutamylation (Figure 5A,B). ICC staining in WT ROs revealed the co-localisation of RPGR and GT335 signals at the CC (yellow signal, Figure 5C), as well as identifying glutamylated tubulin in the CC axoneme (Figure 5C). RPGR-KOs demonstrated a complete lack of RPGR:GT335 co-localisation within the CC (Figure 5C,E), indicating a lack of glutamylated RPGR. AAV-RPGR transduction of RPGR-KO ROs significantly increased GT335 co-localisation with RPGR at the CC (Figure 5C,E). The quantification of GT335:RPGR co-localisation demonstrated significant restoration in transduced ROs (Figure 5E) with overall average RPGR glutamylation levels not significantly different between AAV-RPGR-treated ROs and WT (Figure 5B,E).

RPGR^ORF15^ plays a key role in coordinating the correct trafficking of opsins from the biosynthetic IS to the photoreceptor OS. Previous studies have demonstrated opsin mislocalisation in mouse retina and human ROs [12,20,21]. We quantified rhodopsin mislocalisation in untreated and AAV-RPGR-transduced RPGR-KO ROs by ICC staining. There was a significant accumulation of mislocalised rhodopsin within the photoreceptor cell bodies (ONL) in untreated RPGR-KO ROs when compared to WT ROs (Figure 5D,F). However, AAV-RPGR-treated RPGR-KO ROs demonstrated significantly reduced levels of mislocalised rhodopsin when compared to untreated RPGR-KO ROs, equivalent to levels found within WT ROs (Figure 5D,F).

## 3. Discussion

Variants in the Retinitis Pigmentosa GTPase Regulator (*RPGR*) gene are the dominant cause of XLRP, with the majority of variants located in the retina-specific *RPGR^ORF15^* exon. We sought to establish human in vitro efficacy data for the treatment of *RPGR*-associated XLRP using AAV to deliver a functional copy of *RPGR^ORF15^* to RPGR-deficient human ROs.

Human ROs proffer several unique advantages in the development of gene therapies targeting photoreceptor cells. Here, we used WT human ROs to demonstrate the recapitulation of retinal-tissue-specific mRNA splicing through the upregulation of the *RPGR^ORF15^* isoform, coincidental with retinal development. We generated isogenic *RPGR* variant IPSCs through CRISPR/Cas9 gene editing, which demonstrated pluripotency and showed no retinogenesis deficit, suggesting that RPGR deficiency does not impair retinal precursor differentiation ability, in keeping with other studies [20,30,31]. RPGR-KO ROs were laminated in the inner and outer retinal cells layers and possessed rod and cone photoreceptors with OS structures. Thus, the RPGR-deficient ROs provide us with an excellent in vitro model of our target cell type that is ideally suited to assessing AAV vector potency and vector-derived protein function.

Following the transduction of ROs with the Botaretigene Sparoparvovec clinical construct (utilised in the Phase I/II and Lumeos Phase III clinical trial) packaged into AAV7m8 capsid, robust *RPGR* transgene expression levels were observed. This is in keeping with other published studies employing the RK promoter [12,20,31]. The vector construct produces a protein slightly below the observed molecular weight of WT RPGR^ORF15^, owing to the shortened purine-rich domain of exon 15. This modification has been shown previously to stabilise the repetitive region without compromising RPGR function within photoreceptors [10,12].

Interestingly, within our study we identified the expression of *RPGR* mRNA isoforms within our RPGR-KO ROs, despite the predicted inclusion of PTCs within *RPGR* exon 11. The investigation of this phenotype using RT-PCR highlighted alternative splicing of *RPGR*, specifically the skipping of exon 10, as a plausible cause. The skipping of *RPGR* exon 10, and thus the CRISPR/Cas9-induced 131 bp deletion, avoids the inclusion of premature termination codons within *RPGR* exon 11 and thus enables the expression of *RPGR* mRNA isoforms. Interestingly, CRISPR/Cas9-induced alternative splicing has previously been identified and is believed to be a compensatory mechanism to generate a functionally similar protein to the corresponding full-length protein [32], and is therefore a likely explanation for the retained *RPGR* mRNA levels within the RPGR-KO ROs. Importantly, we detected significantly reduced levels of RPGR protein, suggesting that *RPGR* transcripts lacking exon 10 may produce non-functional protein fragments, which are potentially targeted for protein degradation. Previous studies utilising XLRP patient-derived cell lines harbouring *RPGR* exon 10 variants have also identified exon 10 skipping [25,28], RPGR mislocalisation within the CC [25] and reduced RPGR protein levels [26], further highlighting the importance of *RPGR* exon 10.

RPGR^ORF15^ is known to reside in the photoreceptor CC, where it functions in ciliary gatekeeping, playing a key role in the traffic of proteins between the IS and OS [9]. The photoreceptors within our ROs possess these distinct subcellular compartments (CC, IS, OS), facilitating the functional analysis of our vector-derived RPGR^ORF15^ protein. Analysis of the photoreceptor CC structure demonstrated vector-derived RPGR localised to the apical tip of the ciliary rootlet in a staining pattern that recapitulated the WT isogenic control, in accordance with previously published data [10,12,21,29]. Whilst we also detected some signal in the ciliary rootlet in the RPGR-KO ROs stained with the polyclonal RPGR antibody, this signal was not detected by immunoblotting and we believe this is non-specific cross reactivity with other ciliary-located proteins.

Post-translation glutamylation of the glutamic-acid and glycine-rich domain of RPGR^ORF15^ has been shown to be central to its role within the CC [10], potentially by modifying its affinity for ciliary-interacting partners. The adequate glutamylation of vector-derived RPGR is thus integral to its potency and potential therapeutic function. Using the GT335 polyglutamylation antibody, we were able to demonstrate glutamylation of the vector-derived RPGR^ORF15^ at levels comparable to WT by both immunoblotting and ICC.

Within healthy photoreceptors, the traffic of rod and cone opsin from the biosynthetic IS to their final destination in the OS is essential to proper function. Mislocalisation of the opsin proteins is an early hallmark of photoreceptor degeneration in the RPGR-deficient RD9 mouse model, and the subretinal delivery of AAV-RPGR has been reported to correct cone opsin mislocalisation in vivo [19,20]. Here, we show that rhodopsin is significantly mislocalised to the cell bodies of the ONL in RPGR-KO ROs and demonstrate the successful rescue of rhodopsin trafficking in human photoreceptors following treatment with AAV-RPGR.

In summary, a human photoreceptor cell model of *RPGR*-associated XLRP has been employed to assess the functional potency of the Phase I/II and Lumeos Phase III clinical construct (Botaretigene Sparoparvovec). We demonstrate robust expression of correctly localised and post-translationally modified RPGR^ORF15^, together with the correction of an opsin-mislocalisation phenotype in human ROs. These data provide compelling evidence for a functional vector-derived RPGR protein capable of ameliorating XLRP RPGR-KO phenotypes.

## 4. Materials and Methods

### 4.1. IPSC Generation and Retinal Organoid Differentiation

IPSCs and RPGR-KO IPSCs were generated from commercially available human neonatal dermal fibroblasts (Lonza, Basel, Switzerland, CC-22509). RPGR-KO IPSCs were produced by simultaneous reprogramming and gene editing using a previously described method [33], with minor alterations. Cas9 ribonucleoproteins (RNPs) were designed to target *RPGR* exon 10 using 2 independent guide RNAs (gRNAs), designed and purchased from Synthego (Redwood City, CA, USA). Individual IPSCs were isolated to establish clonal cell lines. IPSC lines were validated for the expression of endogenous self-renewal genes and genotyped by Sanger sequencing using forward and reverse primers targeting *RPGR* exon 10 (Table S1) to confirm the presence of *RPGR* null variants. An IPSC line with a 131 base pair (bp) deletion in *RPGR* exon 10, leading to a predicted PTC in exon 11, was selected for RO differentiation.

RPGR-KO IPSCs and WT IPSCs were differentiated towards a 3D RO fate following previously published protocols [20,34]. At day 135, individual ROs were dosed with 3E11 viral genomes (VGs) of AAV-RPGR. ROs were harvested for analysis 26 days post transduction.

### 4.2. Production of AAV Vector

The RK-RPGR clinical construct comprises the Rhodopsin kinase (RK) promoter and a shortened form of the *RPGR^ORF15^* CDS, comprising a 378 bp in-frame deletion in the linker region of *RPGR* exon 15 [12]. Recombinant AAV7m8 virus was generated by helper virus-free triple transfection. HEK293 suspension cells were transfected with pAAV-hRKp-RPGR, pRep2/Cap7m8 and pHelper at a ratio of 1:1:2. Seventy-two hours after transfection, AAV particles were purified from the clarified lysate by AAVX Affinity chromatography. AAV-containing fractions were concentrated and formulated using a 100 K MWCO protein concentrator using a DPBS buffer supplemented with Pluronic F68 (0.001%). Genomic titres (VG/mL) were determined by QPCR.

### 4.3. RO Processing and Immunocytochemistry

ROs were processed for cryosectioning following either a fixed or unfixed protocol. For fixation, ROs were washed in DPBS, fixed in paraformaldehyde fixative solution (ThermoFisher, Waltham, MA, USA), washed once with DPBS and placed in 30% sucrose solution overnight at 4 °C. The following day, ROs were embedded in Optimal Cutting Temperature (OCT) compound before freezing at −80 °C. Organoids were sectioned at 6 µm and stored at −20 °C. For unfixed processing, ROs were washed in DPBS and embedded in OCT before freezing at −80 °C. Unfixed RO sections were treated with 0.5% PFA for 10 min at room temperature (RT) before further processing.

RO sections were stained with primary antibodies (Table S2) overnight at 4 °C, before staining with Alexafluor 488 or 555 secondary antibodies (ThermoFisher). Cell nuclei were identified with 4′,6-diamidino-2-phenylindole (DAPI, Sigma, St. Louis, MO, USA).

### 4.4. RNA Extraction, QPCR and RT-PCR

Total RNA was extracted from ROs using the PicoPure RNA Isolation Kit (Arcturus, San Diego, CA, USA). First strand cDNA synthesis was performed using the Superscript IV Vilo Master Mix (ThermoFisher) using 200 ng of total RNA per sample.

QPCR analysis was performed using TaqMan Fast Advanced Master Mix (ThermoFisher) following standard cycling parameters using TaqMan assays to identify genes of interest (Table S3). Primers and probes targeting *RPGR^ORF15^* were designed using the IDT PrimerQuest Tool (https://eu.idtdna.com/PrimerQuest/Home, accessed on 2 February 2022) to specifically amplify the *RPGR^ORF15^* transcript only (Integrated DNA Technologies, Coralville, IA, USA; Table S3). The mRNA levels for target genes were normalised to the geometric mean of endogenous reference genes *hGAPDH* and *β-ACTIN* and expressed as relative expression versus WT ROs. QPCR statistical analysis was completed on delta delta cycle threshold (Ct) values.

RT-PCR was completed to determine the alternative splicing of *RPGR* exon 10. Primers were designed in PrimerBLAST (Table S1), focusing on the exons surrounding *RPGR* exon 10. RT-PCR was completed using standard PCR cycling procedures, using Platinum SuperFi II DNA polymerase (ThermoFisher) and 1 µL cDNA per reaction. RT-PCR reactions were analysed on a 1% agarose gel containing DNA SYBR Safe and imaged on a Biorad ChemiDoc MP (Biorad, Hercules, CA, USA).

### 4.5. Protein Extraction and Immunoblotting

RO samples were lysed in radioimmunoprecipitation buffer containing 1% protease inhibitor cocktail (ThermoFisher) on ice followed by sonication. Total protein quantification was completed using the Pierce Bicinchoninic Acid (BCA) Protein Assay (ThermoFisher). Protein immunoblotting was completed using the Wes automated Western blot system using antibodies listed in Table S2. Protein expression was normalised to internal loading controls and expressed as relative expression versus WT samples. RPGR glutamylation levels were expressed as a ratio of GT335 to β-tubulin.

### 4.6. Imaging and Image Analysis

Confocal images were acquired using a Leica SP8 confocal microscope (Leica, Wetzlar, Germany). Maximum intensity projections were created from Z stacks and utilised for downstream analysis.

RO RPGR-GT335 co-localisation and ONL rhodopsin mislocalisation were quantified using FiJi image processing software (ImageJ, version 2.14.0/1.54f). The quantification of RPGR-GT335 co-localisation was performed on the peripheral region of the RO, identified as the region outside of the ONL containing both RPGR and GT335 staining. The region of interest (ROI) was selected, and colour channels split. The ROI was applied to individual channels and the area outside the ROI cleared. Subsequently, a threshold was applied to each channel, which were all set and maintained for each imaging set. Co-localisation between imaging channels was identified using the FiJi “colocalisation plugin”. For the quantification of ONL rhodopsin mislocalisation, the ROIs containing the ONL only (DAPI positive cells) and ONL with POS (DAPI-positive cells with RHODOPSIN-positive outer segments) were identified, selected and transferred individually to the rhodopsin channel. The area outside the ROI was cleared and a threshold applied to the selected ROI. Images were quantified as “pixels above threshold” and expressed as RHODOPSIN-positive pixels in the ONL against total RHODOPSIN-positive pixels (%).

### 4.7. Data Processing

Statistical analysis was completed using GraphPad Prism 9 software (Version 9.3.1; GraphPad Inc.) using an ordinary one-way ANOVA with Tukey’s multiple comparison test or two-way ANOVA with a Sidak’s multiple comparison test. Results are represented as arithmetic mean with upper and lower SEM.

## Data Availability

Data is contained within the article and Supplementary Materials.

## Supplementary Materials

The following supporting information can be downloaded at: https://www.mdpi.com/article/10.3390/ijms25031839/s1.
