# Supplementary material for: AAV-RPGR Gene Therapy Rescues Opsin Mislocalisation in a Human Retinal Organoid Model of RPGR-Associated X-Linked Retinitis Pigmentosa
Source: Int J Mol Sci. 2024 Feb 2;25(3):1839. doi: 10.3390/ijms25031839 (PMC10855600; doi:10.3390/ijms25031839)
Supplement: Supplementary file 1 [file ijms-25-01839-s001.zip › ijms-2784418-supplementary.pdf]

Supplementary Table S1. RPGR genotyping and RT-PCR primers.

Gene target	Forward	Reverse
RPGR exon 10 genotyping	GAGACATAATTAGCTCAAGAGACTG	AGTGAAAGTCAGTCCACATGC
RPGR exon 10 alternative splicing	GGTCGCCACGGAAAATTAGG	TCAGTAGTTTCTCCAAGGCTTTCT

Supplementary Table S2. Antibodies used for immunocytochemistry and WES analysis.

Antibody target	Manufacturer	Catalogue number	Use	Dilution (ICC/WES)
β-tubulin	Abcam	Ab21058	WES	1:500,000
CRX	Abnova	H00001406-M02	ICC	1:1000
GT335	Adipogen	AG-20B-0020-C100	ICC/WES	1:200/1:1000
L/M Opsin	Merck Millipore	AB5405	ICC	1:500
RECOVERIN	Merck Millipore	AB5585	ICC	1:500
Rhodopsin	Merck Millipore	MABN15	ICC	1:500
ROOTLETIN	Santa Cruz	SC-374056	ICC	1:200
RPGR	Sigma	HPA001593	ICC/WES	1:500/1:40

Supplementary Table S3. QPCR TaqMan assays utilised for RO gene quantification.

Gene	ThermoFisher TaqMan Assay ID	Stock concentration
ARR3	Hs01020134_m1	900nm (primer), 200nm (probe)
β*ACTIN*	Hs01060665_g1	900nm (primer), 200nm (probe)
GAPDH	Hs02758991_g1	900nm (primer), 200nm (probe)
GNAT2	Hs00292542_m1	900nm (primer), 200nm (probe)
L/M OPSIN	Hs04194752_g1	900nm (primer), 200nm (probe)
NR2E3	Hs00183915_m1	900nm (primer), 200nm (probe)
NRL	Hs00172997_m1	900nm (primer), 200nm (probe)
Rhodopsin (RHO)	Hs00892431_m1	900nm (primer), 200nm (probe)
RECOVERIN	Hs00975544_m1	900nm (primer), 200nm (probe)
$RPGR^{1-19}$	Hs01553405_m1	900nm (primer), 200nm (probe)
S-OPSIN	Hs00181790_m1	900nm (primer), 200nm (probe)

Gene	Forward	Reverse	Probe	Concentrations
$RPGR^{ORF15}$	GCTACGACTATC GAAGCATTT	TCTGCTTCTCC CACTGATT	ACAAAGCAGAGG TGAGTGAAGGCA	500nm (primer), 250nm (probe

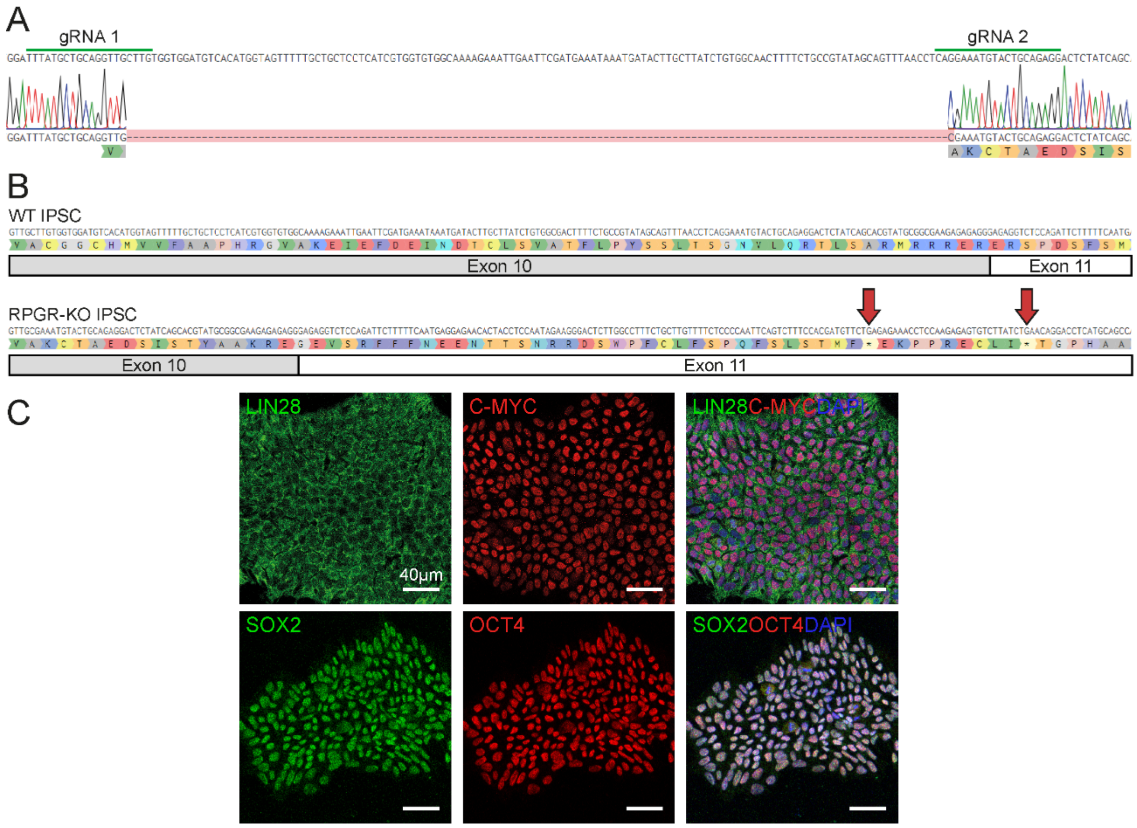

Figure S1. Generation and characterisation of RPGR-KO IPSC (**A**) RPGR-KO IPSCs were generated using simultaneous reprogramming and CRISPR/Cas9 gene editing by targeting *RPGR* exon 10 with 2 guide RNAs (gRNA; green bars), inducing a 131 base pair deletion, confirmed by Sanger Sequencing. (**B**) Predicted effects of *RPGR* exon 10 editing, causing downstream frameshift and premature termination codons (red arrows) in exon 11. (**C**) IF analysis confirmed expression of ESC markers OCT4, LIN28, C-MYC and SOX2 in RPGR-KO IPSCs. Nuclei are identified with DAPI (blue). Scale bar = 40 µm.

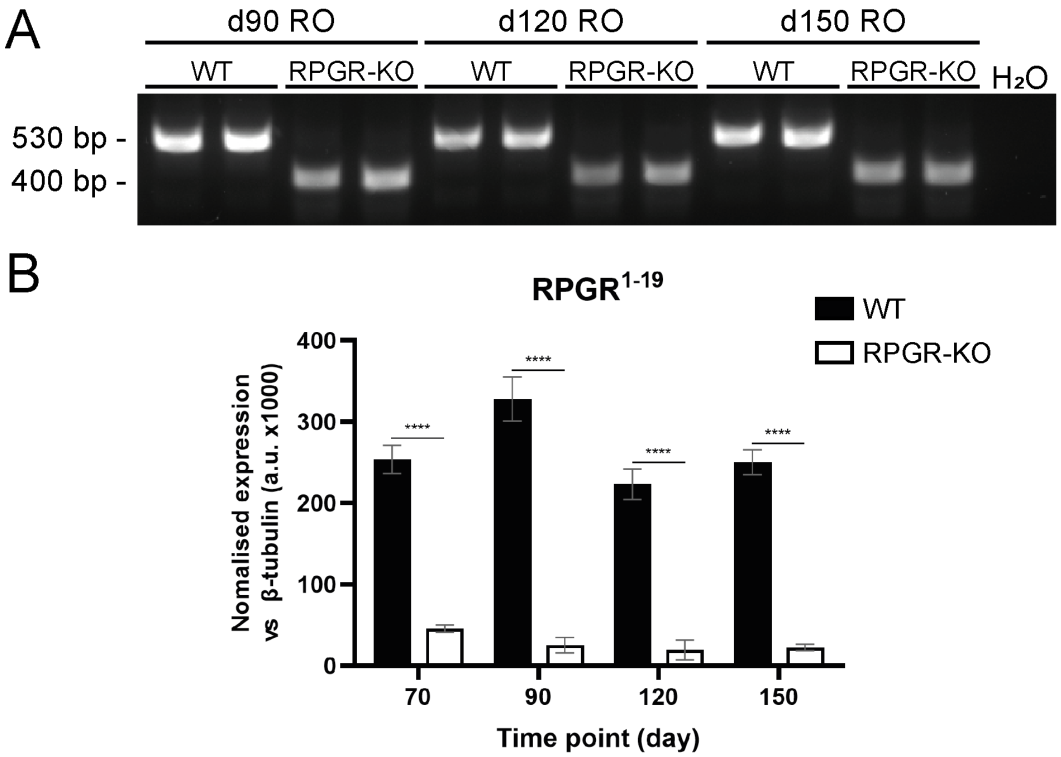

Figure S2. Analysis of *RPGR* exon skipping and RPGR$^{1-19}$ protein isoform. (A) RT-PCR analysis of *RPGR* exon skipping at d90, d120 and d150 in WT and RPGR-KO ROs. Primers were designed to target alternative splicing of *RPGR* exons 9, 10, 11 and 12. RPGR-KO ROs demonstrate exon skipping of *RPGR* exon 10, resulting in a smaller PCR product (400bp) when compared to WT ROs (530bp). H_2O = no cDNA control. **(B)** WES quantification demonstrates significant reduction of RPGR$^{1-19}$ protein in RPGR-KO ROs when compared to WT ROs. n=2-4 ROs, **** $p<0.0001$.
